# Faecal volatile biomarkers of *Clostridium difficile* infection

**DOI:** 10.1371/journal.pone.0215256

**Published:** 2019-04-15

**Authors:** Mitesh Patel, Dawn Fowler, Jeremy Sizer, Christopher Walton

**Affiliations:** 1 School of Water, Energy and Environment, Cranfield University, Cranfield, United Kingdom; 2 Bedford Hospital NHS Trust, South Wing, Bedford, United Kingdom; Cornell University, UNITED STATES

## Abstract

Care of patients with potential CDI can involve isolation and use of antibiotics, often before a definitive diagnosis is available, impacting healthcare resource and contributing to antibiotic resistance. There is anecdotal evidence that the faeces of CDI patients have a distinctive odour, while it is well-established that changes in the gut microbiota are associated with changes in the volatile organic compounds (VOC) produced. A total of twenty-four candidate volatile biomarkers were identified from a review of the literature including *in vitro*, animal and human studies. Using thermal desorption-gas chromatography-time-of flight mass spectrometry (TD-GC-ToFMS), VOC emission rates were determined on stored frozen stool samples from 53 CDI-positive and 53 CDI-negative patients with unexplained diarrhoea which had previously been diagnosed using enzymatic and nucleic acid amplification tests. Sample preparation was limited to placement of a subsample in an appropriate container. Compounds exhibiting a statistically significant difference (p < 0.05) in emission rate between the CDI-positive and–negative groups and a corresponding area under the receiver-operator characteristic curve (ROC) >0.7 were considered potentially indicative of CDI. Seven compounds were so identified: propan-1-ol (ROC 0.75), 3-methylbutanal (ROC 0.84), ethyl propionate (ROC 0.81), hexanoic acid (ROC 0.73), 4-methylphenol (ROC 0.81), dodecane (ROC 0.80) and indole (ROC 0.85). A number of potential volatile biomarkers of CDI can be sampled rapidly and with little prior preparation from faecal samples of patients with diarrhoea. Of these 4-methylphenol (*p*-cresol) is of particular interest as it has been anecdotally linked to CDI and is closely related to the biology and virulence of *Clostridium difficile*. This approach shows promise for the rapid, point-of-care diagnosis of CDI with good sensitivity and specificity.

## Introduction

*Clostridium difficile* infection (CDI) can produce symptoms ranging from mild to severe diarrhoea to pseudomembranous colitis. Prior exposure to antibiotics is a major predisposing factor and those unable to mount a strong immune response, particularly the elderly, are most vulnerable. Severe cases can be fatal. Although considered to be usually a nosocomial infection, there is evidence that CDI is becoming more prevalent in communal settings such as prisons and care homes [1]. CDI is associated with increased length of hospital stay and significant mortality with a concomitant drain on healthcare resources. There is limited information on the economic impact of CDI, though significant attributable costs have been estimated for both Europe and the US, with higher costs associated with recurrent CDI [2, 3].

The worldwide incidence of hospital-acquired CDI is significant, driven by epidemiological factors and the emergence of hypervirulent strains exhibiting both antibiotic resistance and increased toxin production [4]. In the UK, incidence fell dramatically between 2007 and 2015 as a result of improved housekeeping measures by NHS Trusts and a modified antibiotic prescribing policy. Figures for England show an incidence of over 16,864 for all reported cases in the second quarter of 2007 diminishing to 3,388 by the first quarter of 2015. However, *Clostridium difficile* is carried as a commensal gut bacterium within the population so that a reservoir of infection exists. In this context it is notable that UK incidence has recently remained stable and it seems that significant further reductions are unlikely.

Clinical diagnosis of CDI is based on enzymatic immunoassay (EIA) and nucleic acid amplification tests (NAAT). EIAs are targeted against glutamate dehydrogenase (GDH) and bacterial toxins A and B, while NAAT are targeted against the genes associated with toxin production. Toxin EIAs are generally acknowledged to have poor sensitivity (values as low as 40% have been reported in trials against culture-based reference methods [5]), their main value lying in their low rate of false negative results. GDH assays and NAAT, conversely, have relatively high sensitivity but respond to the presence of the organism rather than its virulence so cannot distinguish carriage of the *Clostridium difficile* bacterium from CDI.

These limitations have resulted in the development of diagnostic pathways where more than one class of test is used to establish a diagnosis of CDI [6]. The current UK NHS guidance specifies the use of a GDH assay in conjunction with either a toxin EIA or NAAT [7]. These tests can deliver results in minutes, but are nonetheless laboratory-based so that the effective request-to-result time is more commonly measured in hours. Because of this, patients with unexplained diarrhoea may be prescribed antibiotic therapy and placed in isolation before the results become available. This places an additional burden on resources as well as potentially contributing to the spread of antibiotic resistance. There is therefore a clear clinical need for tests with a shorter time-to-result and sufficient specificity to exclude CDI at an early stage.

There is anecdotal evidence that stool from CDI patients has an identifiable odour, suggesting that detection of odorants might offer a means of rapid, point-of-care diagnosis. Analysis of volatile organic compounds (VOCs) released from faecal samples has revealed profiles associated with a range of gastrointestinal disorders [8–13], while reports going back several decades have suggested specific VOCs as potential markers for CDI [10, 11, 14–23]. However, these studies have used methods involving culturing *Clostridium difficile*, some degree of prior sample preparation and/or prolonged sampling times. Although these findings are promising, in order to develop a practicable, rapid diagnostic method it is important to establish the extent to which VOCs from stool having little or no preparation and sampled for only a short time can be associated with disease. In this study, we have investigated stool samples obtained from patients with diarrhoea who were diagnosed as either positive or negative for CDI using standard clinical techniques. We have applied the method of thermal desorption–gas chromatography–mass spectrometry (TD-GC-MS) which allows active pre-concentration of VOCs onto a solid sorbent and their subsequent determination using a targeted metabolomics approach. We used a relatively short sampling time (5 min), with sample preparation being limited to placing it in an appropriate container.

## Materials and methods

### Target compound selection

Compounds were selected for quantitative analysis on the basis of an examination of the relevant literature. This comprised reports dealing with the metabolism of *C difficile in vitro* [14, 18, 21, 23, 24], animal studies [16] and studies of faecal biomarkers in human volunteers [10, 11, 19]. Additionally, several compounds were included on the basis of an initial visual inspection of the chromatographic data. Twenty-four compounds were selected in total. **Table 1** lists the target compounds and indicates the basis for the inclusion of each.

**Table 1:** VOC emission rates. Emission rates of VOCs in headspace of faecal samples referred for testing for Clostridium difficile and subsequently classified as either positive or negative by a combination of glutamate dehydrogenase, nucleic acid and enzymatic tests. References are given for the source material relevant to the inclusion of each compound, where no reference is given the compound was included on the basis of initial visual inspection of the data.

				Median emission rate (ng.l^-1^.s^-1^) (lower quartile, upper quartile % non-detects)			
Compound (IUPAC)	Other names	CAS No.	RT (min)	Negative (N = 53)	Positive (N = 53)	*P*	ROC (95% lower, upper confidence bounds)
Propan-1-ol [10]		71-23-8	7.164	44.26	249.45	<0.0005	0.746
				(0.00, 121.18)	(88.44, 461.88)		(0.652, 0.841)
				38%	11%		
Butan-2-one [10]	Methyl ethyl ketone	78-93-3	8.600	10.28	7.61	0.162	0.579
				(4.56, 28.31)	(4.40, 13.38)		(0.469, 0.689)
				2%	0%		
2-methylpropan-1-ol [10]		78-83-1	9.500	3.84	6.99	0.033	0.620
				(1.99, 8.58)	(2.68, 17.27)		**(0.512, 0.728)**
				11%	13%		
3-methylbutanal	Isovaleraldehyde	590-86-3	10.207	1.18	2.69	<0.0005	0.837
				(0.95, 1.53)	(1.64, 4.63)		(0.762, 0.912)
				9%	2%		
Butan-1-ol [23]		71-36-3	11.000	15.31	15.97	0.615	0.472
				(11.32, 22.41)	(4.29, 36.53)		(0.355, 0.588)
				0%	11%		
1-Penten-3-ol [10]		616-25-1	11.493	1.38	1.39	0.454	0.542
				(1.29, 1.44)	(1.26, 1.57)		(0.431, 0.653)
				13%	11%		
Ethyl propionate [10]		105-37-3	13.075	0.00	7.25	<0.0005	0.805
				(0.00, 2.02)	(1.86, 86.15)		(0.721, 0.888)
				53%	11%		
Methyl butanoate [10]	Butyric acid methyl ester	623-42-7	14.000	0.00	0.00	0.065	0.582
				(0.00, 0.00)	(0.00, 0.14)		(0.473, 0.691)
				79%	66%		
3-methyl-1-butanol [10]		123-51-3	14.800	10.55	11.80	0.942	0.496
				(5.48, 25.11)	(4.99, 21.38)		(0.385, 0.607)
				2%	0%		
1*H*-Pyrrole [10]	Pyrrole	109-97-9	15.611	0.69	0.70	0.153	0.578
				(0.00, 0.72)	(0.00, 0.75)		(0.469, 0.687)
				45%	32%		
Furan-2-carbaldehyde [11]	Furfural	98-01-1	21.099	0.00	0.00	0.304	0.460
				(0.00, 0.00)	(0.00, 0.00)		(0.350, 0.571)
				77%	85%		
3-Methylbutanoic acid [18]	Isovaleric acid	503-74-2	21.469	0.00	1.91	<0.0005	0.701
				(0.00, 0.00)	(0.00, 5.11)		(0.600, 0.801)
				75%	43%		
2-Methylbutanoic acid [10, 16]		116-53-0	22.272	0.00	0.00	<0.0005	0.663
				(0.00, 0.00)	(0.00, 7.87)		(0.558, 0.767)
				87%	58%		
Heptan-4-one [10]		123-19-3	23.770	0.00	0.88	<0.0005	0.714
				(0.00, 0.00)	(0.00, 1.18)		(0.616, 0.813)
				75%	40%		
4-Methylpentanoic acid [15, 16, 18–20, 22]	Isocaproic acid	646-07-1	27.827	1.65	2.21	0.005	0.652
				(0.00, 2.42)	(0.00, 7.95)		(0.547, 0.757)
				49%	32%		
5-methyl-2-furancarboxaldehyde [11]	5-methylfurfural	620-02-0	28.842	ND	ND		
Hexanoic acid [10, 17, 19]	Caproic acid	142-62-1	29.311	3.16	2.42	<0.0005	0.730
				(2.71, 3.81)	(0.00, 3.01)		(0.633, 0.828)
				38%	9%		
Decane		124-18-5	30.357	0.69	0.69	0.651	0.525
				(0.63, 0.77)	(0.65, 0.73)		(0.413, 0.638)
				0%	0%		
2-(2-Ethoxyethoxy) ethanol [10]	Transcutol	111-90-0	30.802	0.00	0.00	0.690	0.489
				(0.00, 0.00)	(0.00, 0.00)		(0.379, 0.600)
				91%	92%		
4-Methylphenol [17, 21, 22]	*para*-cresol *p-cresol*	106-44-5	33.489	7.99	23.68	<0.0005	0.814
				(5.21, 13.36)	(14.71, 42.38)		(0.729, 0.899)
				0%	0%		
Dodecane		112-40-3	37.455	0.91	0.86	<0.0005	0.803
				(0.90, 0.95)	(0.83, 0.90)		(0.715, 0.890)
				0%	2%		
Indole [11, 14]		120-72-9	39.824	1.57	4.24	<0.0005	0.852
				(1.29, 1.93)	(2.55, 7.94)		(0.773, 0.931)
				0%	2%		
3-Methylindole [10]	Skatole	83-34-1	41.580	1.18	1.10	0.626	0.527
				(1.05, 1.41)	(1.05, 1.43)		(0.415, 0.640)
				17%	13%		
Pentadecane		629-62-9	42.958	0.86	0.84	0.006	0.654
				(0.84, 0.89)	(0.81, 0.88)		(0.547, 0.760)
				0%	2%		

P indicates statistical significance by Mann Whitney U test. ROC = area under receiver-operator characteristic curve. RT = chromatographic retention time. ND = not detected in any sample.

### Sampling

Frozen (-40°C) faecal samples were obtained from the infection control laboratory at St Thomas’ Hospital NHS Trust, London, UK. These samples had previously been tested for *Clostridium difficile* using glutamate dehydrogenase (GDH) assays, nucleic acid amplification tests and enzymatic assays for bacterial toxins. Fifty-three positive and 53 negative samples were selected by staff of the infection control laboratory. Data were completely anonymised so that patient consent was not required. Ethical approval precluded collection of metadata. This study was approved by the National Research Ethics Service Committee East of England–Hertfordshire (REC reference: 11/EE/0095) and by the Cranfield University Health Research Ethics Committee (Ref No. 12/11).

**Fig 1:**
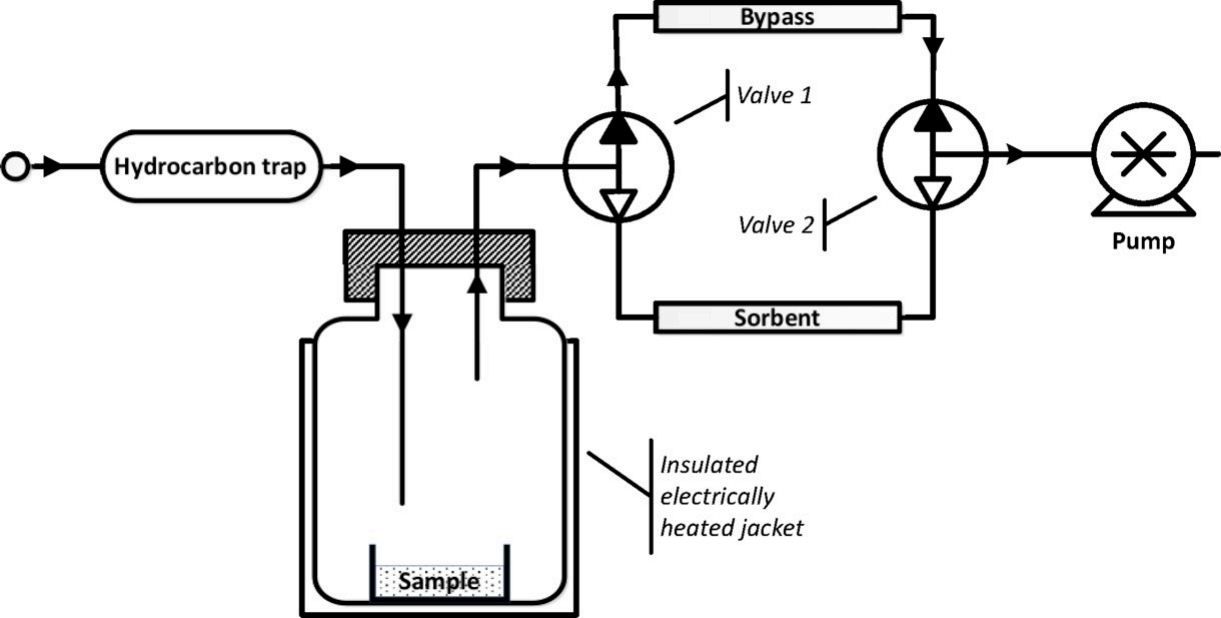
Apparatus for sampling faecal volatiles onto thermal desorption (TD) tubes. The sample is placed in a temperature-controlled bottle under a constant flow of air from which hydrocarbons have been trapped. After equilibration, airflow is diverted through a TD tube which retains volatile organic compounds emitted by the sample and which is then removed for subsequent laboratory analysis.

The sampling campaign was carried out at the infection control laboratory using portable equipment. Samples were thawed at room temperature while remaining sealed in their original containers. A single subsample was then taken and placed in an open container. Vial caps were used for this purpose in order to ensure that each sample had a similar free surface area and volume. The container was placed on the bottom of a 100 ml Duran bottle which was closed by a lid having ports to allow air to be drawn through (**Fig 1**). The bottle was thermally insulated and was electrically heated to maintain a temperature of 40°C. The sample was left to equilibrate for 10 min with air being drawn through at a constant rate of 100 ml.min^-1^ using a portable pump (Gilair Plus, Shawcity Ltd, Watchfield, UK). Before reaching the sample, air was passed through an activated carbon trap to remove atmospheric hydrocarbons. After equilibration, the flow of air was diverted to pass through a thermal desorption (TD) sampling tube containing Tenax TA and Carbotrap sorbents (50:50) (Markes International Ltd, Llantrisant, UK). Sampling time was 5 minutes for a sample volume of 500 ml. Temperature and timing were controlled using an Arduino open-source microcontroller. To avoid cross-contamination, separate sets of sampling equipment were used for positive and negative samples. TD tubes were immediately capped following sampling and were returned each day to Cranfield University for analysis.

### Laboratory analysis

An internal standard solution containing 50 ng d8-toluene (Supelco Cat No. 48593) in methanol was added to each thermal desorption tube using a calibration solution loading rig according to the manufacturer’s instructions (CSLR, Markes International Ltd, Llantrisant, UK). Headspace samples were analysed by GC-ToFMS. Analytical instrumentation comprised a Series 2 Unity thermal desorber with Series 2 Ultra autosampler, an Agilent model 7890 gas chromatograph and ALMSCO Bench-ToF-GC-MS (Markes International, Llantrisant, UK). The gas chromatograph was fitted with a DB5 column of dimensions 60m x 0.4mm x 0.25mm (Agilent Technologies UK Limited, Stockport, UK). The carrier gas was CP grade helium (BOC gases, Guildford, UK) passed through a combined trap for removal of hydrocarbons, oxygen and water vapour.

TD tubes were purged for 1 minute in order to remove air and water vapour then desorbed at 300°C for 8 minutes onto the secondary cold trap (Materials emission trap, U-T12ME-2S Markes International Ltd, Llantrisant, UK) which was initially maintained at -10°C. Once desorption was complete, the secondary trap was heated at the maximum available rate to 300°C and maintained for 3 minutes whilst the effluent was transferred to the GC via a transfer line maintained at 150°C. Constant-flow operation was used, at 1.2 ml min^-1^. Initial GC temperature was 35°C held for 1 minute, then increased at 2°C /min to 75°C, 5°C/min to 140°C and 10°C/min to 300°C where it was held for 10 minutes. The eluted products were transferred to the MS via a transfer line maintained at 200°C where they were subjected to electron ionisation, the ion source temperature being maintained at 200°C. The MS was operated from 33–480 amu at an effective scan rate of approximately 2.2 Hz.

Compound identification and quantification was achieved using Chemstation (G1701EA Revision E.02.01), Automated Mass spectral Deconvolution and Identification System (AMDIS) Version 2.62 and the National Institute of Standards and Technology (NIST) mass spectral library. The system was calibrated using analytical grade standards of the target compounds dissolved in methanol and added to TD tubes using the CSLR. Response curves against the internal standard were then generated using Chemstation. Calibration was repeated as necessary, for example following tuning of the mass spectrometer.

### Data analysis

Results were processed using IBM SPSS Version 22. Data quantified using Chemstation as mass of each analyte per TD tube were converted to emission rates using the known headspace sample volume and sampling time. Differences between CDI-positive and CDI-negative groups were assessed using the Mann-Whitney U Test for unpaired samples. ROC curves were prepared using SPSS functionality which allows the sense of the resulting curve to be changed. This was done for compounds with a lower median emission rate in CDI-positive than CDI-negative samples in order to give all ROC curves the same sense and aid visual comparison.

### Controls

VOC emission from the apparatus was assessed by drawing samples through an empty bottle. This was done for each set of apparatus at least once on each day of sampling for a total of 16 samples. The VOC content of laboratory air was determined by drawing samples (N = 5 at the same flow rate and duration) through a TD tube connected directly to a pump. Finally, the contribution of the analytical instrumentation was monitored by the inclusion of a TD tube bearing internal standard alone in each analytical run (N = 15).

## Results

Data analysis was carried out using SPSS (version 22, IBM UK Ltd, Portsmouth, UK). Data were expressed for each compound as rate of release per unit volume of the sample (ng.l^-1^.min^-1^). Median, upper quartile and lower quartile emission rates for *C difficile* positive and negative samples are given in **Table 1**. Emission rates for most compounds examined were found to follow a non-normal frequency distribution and moreover a number of non-detects were recorded, therefore differences between emission rates from positive and negative samples were examined using the non-parametric Mann-Whitney U test for unpaired samples. Non-detects were treated as zeros and were included in the analysis. Percentages of non-detects for each compound are indicated in **Table 1**. Receiver operating characteristic (ROC) curves were calculated for each compound and the areas under the curves (AUC) are shown in **Table 1** with their 95% confidence bounds.

**Table 2** shows the equivalent emission rates for the control samples. The target compounds were not detectable in a majority of the internal standard analyses and where present were at much lower abundance than the test samples. This was generally true for the empty bottle controls but with several exceptions. Most striking was 1-penten-3-ol which appeared to be much more abundant in the control than the test samples. It is possible that this is the result of contamination from the ambient air, but is considered to be more likely due to the difficulty of positively identifying this compound by its mass spectral signature in our dataset. The median abundance of methyl butanoate was higher in the control than the test samples, but it was in any case not detectable in the majority of cases. Similarly 1H-pyrrole was similar in both abundance and the proportion of non-detects between test and control samples. The abundance of the target compounds was in most cases higher in the ambient air samples than the other controls, indicating that the use of a hydrocarbon trap to provide a flow of clean air for sampling proved effective. Again there were some exceptions to this (butan-1-ol, 1-penten-3-ol, methyl butanoate, 1h-pyrrole, 4-methylphenol), suggesting that a small amount of carryover or contamination occurred. However as outlined above this is unlikely to have materially affected the results.

**Table 2:** Emission rates from controls. Equivalent emission rates of faecal headspace VOCs determined in control samples.

Compound (IUPAC)	IAQ	Empty bottle	Internal standard only
	Equivalent median emission rate	Equivalent median emission rate	Equivalent median emission rate
	(ng.l^-1^.s^-1^)	(ng.l^-1^.s^-1^)	(ng.l^-1^.s^-1^)
	(lower, upper quartile	(lower, upper quartile	(lower, upper quartile
	% ND)	% ND)	% ND)
	N = 5	N = 16	N = 15
Propan-1-ol	ND	ND	ND
Butan-2-one	4.69	0.00	0.00
	(3.28, 5.06)	(0.00, 0.00)	(0.00, 0.14)
	0%	88%	73%
2-methylpropan-1-ol	1.35	0.00	0.00
	(0.00, 1.88)	(0.00, 1.28)	(0.00, 1.25)
	40%	56%	67%
3-methylbutanal	0.00	0.00	0.00
	(0.00, 0.00)	(0.00, 0.62)	(0.00, 0.00)
	80%	69%	87%
Butan-1-ol		1.74	0.00
	ND	(0.00, 3.72)	(0.00,0.00)
		44%	87%
1-Penten-3-ol	3.25	11.12	0.00
	(3.11, 3.48)	(0.69, 11.77)	(0.00, 1.29)
	0%	25%	53%
Ethyl propionate	0.00	0.00	
	(0.00, 0.00)	(0.00, 1.14)	ND
	80%	69%	
Methyl butanoate		0.69	0.00
	ND	(0.00, 0.76)	(0.00, 0.73)
		44%	67%
3-methyl-1-butanol	0.00	0.00	0.00
	(0.00, 0.00)	(0.00, 0.00)	(0.00, 0.00)
	80%	94%	93%
1*H*-Pyrrole		0.68	0.00
	ND	(0.00, 0.70)	(0.00, 0.62)
		44%	73%
Furan-2-carbaldehyde	2.15		
	(0.00, 2.34)	ND	ND
	40%		
3-Methylbutanoic acid	0.00	0.00	
	(0.00, 3.62)	(0.00, 0.65)	ND
	60%	75%	
2-Methylbutanoic acid	0.00	0.00	0.00
	(0.00, 0.00)	(0.00, 0.00)	(0.00, 0.00)
	80%	88%	93%
Heptan-4-one		0.00	
	ND	(0.00, 0.00)	ND
		88%	
4-Methylpentanoic acid	5.35	0.00	
	(5.12, 8.33)	(0.00, 0.00)	ND
	20%	88%	
5-methyl-2-furancarboxaldehyde			
	ND	ND	ND
		
Hexanoic acid	8.27	1.12	0.00
	(6.01, 11.41)	(0.00, 3.12)	(0.00, 0.00)
	20%	50%	93%
Decane	2.77	0.00	0.00
	(2.62, 4.82)	(0.00, 0.01)	(0.00, 0.50)
	0%	75%	73%
2-(2-Ethoxyethoxy) ethanol	ND	ND	ND
4-Methylphenol	0.00	0.86	0.00
	(0.00, 0.00)	(0.16, 2.41)	(0.00, 0.00)
	80%	25%	93%
Dodecane	1.42	0.00	0.00
	(1.25, 1.56)	(0.00, 0.00)	(0.00, 0.68)
	20%	88%	67%
Indole	0.00	0.18	0.00
	(0.00, 0.96)	(0.00, 0.44)	(0.00, 0.00)
	60%	31%	93%
3-Methylindole		0.00	
	ND	(0.00, 0.01)	ND
		75%	
Pentadecane	1.17	0.00	0.00
	(1.12, 1.23)	(0.00, 0.00)	(0.00, 0.79)
	20%	88%	67%

IAQ = Indoor Air Quality (Laboratory air) sample. Empty bottle = headspace sampling system operated without sample. Internal standard only = Blank thermal desorption tube loaded with d8-toluene internal standard and analysed. % indicates percentage of non-detects; ND = not detected in any sample.

Compounds having the highest potential as biomarkers for CDI were selected using three criteria: First, a statistically significant difference (p < 0.05) in emission rate between CDI-positive and CDI-negative samples; second, an area under the ROC curve >0.7 and third a measurable presence in at least 80% in at least of one of the two groups. The latter, fairly arbitrary, criterion was introduced in view of the high proportion of non-detects observed with some compounds (e.g. 3-methylbutanoic acid) where a significant difference and high AUC were found but which was not detectable in a high proportion of samples using our current method (75% and 43% in this example for CDI-negative and -positive respectively). Seven compounds met all three criteria: propan-1-ol, 3-methylbutanal, ethyl propionate, hexanoic acid, 4-methylphenol, dodecane and indole. Because of its close relationship with virulence (see Discussion) 4-methylphenol (*para*-cresol) is of particular interest.

**Fig 2:**
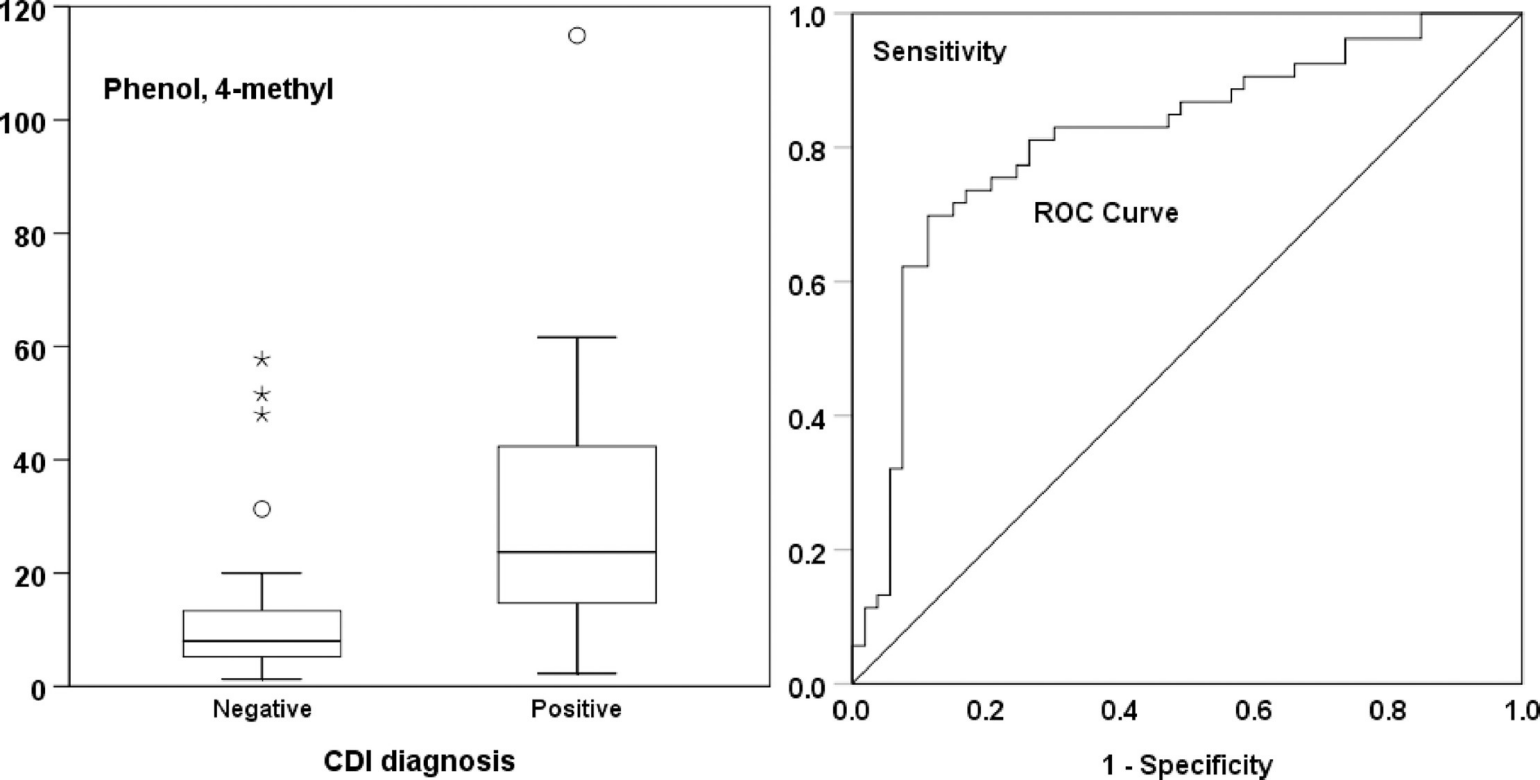
Box-and-whisker plot and corresponding receiver-operator characteristic (ROC) curve. These plots relate to the emission rate of 4-methylphenol (para-cresol) from faecal samples diagnosed as negative or positive for Clostridium difficile infection (CDI) using molecular techniques. Box plot: horizontal bar indicates the median; boxes include the first to third quartiles, whiskers show the 5% and 95% centiles and outliers are indicated by individual symbols.

**Fig 2** the sense shows a box plot of emission rate from CDI-negative and–positive samples and the corresponding ROC curve.

## Discussion

In this study, we have investigated the potential application of faecal VOC analysis for the rapid diagnosis of CDI. In order to identify potential volatile biomarkers for CDI we adopted a targeted approach focussing on twenty-four compounds which had been previously reported as being associated with CDI, produced by *Clostridium difficile in vitro*, or which appeared discriminatory from an initial visual inspection of the TD-GC-MS data. For convenience these compounds can be considered as falling into several broad groups: short-chain fatty acids (SCFAs) and their derivatives, ketones, aromatics and alkanes. Although the overall constitution of VOCs in the gut is influenced by diet and host metabolic processes, there is for the majority of these compounds a plausible pathway for their production by the gut microbiota. SCFAs are produced by bacterial fermentation of dietary polysaccharides [25] while it has been shown that these in turn can be metabolised to their homologous branched derivatives, aldehydes and esters by cultured human stool samples [10]. Ketones and alkanes are probably produced through lipolysis, while aromatics originate from the catabolism of aromatic amino acids [14]. Applying a set of empirical criteria to our data, we identified seven potential biomarkers for CDI from an initial candidate list of twenty-four.

A number of methodological approaches have been used to investigate the utility of faecal VOCs for the diagnosis of CDI. Solid-phase micro-extraction (SPME) with analysis by GC-MS has been applied to a range of gastrointestinal disorders including inflammatory bowel disease [8], irritable bowel syndrome [9], CDI [10] and some less common conditions, for example Giardiasis [26]. Using this technique, a fibre coated in an adsorbent is extruded into the headspace of the sample, allowed to equilibrate and then desorbed at high temperature for analysis using an essentially conventional GC injector. In this application, a very wide range of compounds can be detected and one approach has been to score these (sometimes several hundred in number, see [10]) according to their presence or absence in any given sample. Multivariate analysis of the resulting scores has been very successful in discriminating a range of conditions. Results from SPME extraction are most reliable if concentrations in the fibre and headspace are allowed to reach equilibrium. The time required for this depends upon the type and thickness of the adsorbent coating of the fibre as well as the distribution constants of individual VOCs. It has been noted that prolonged (up to several hours) extraction times and the use of a range of adsorbent coatings dramatically increases the number of VOCs that can be detected [27, 28]. This approach is therefore well suited to characterisation of the faecal volatilome but is probably of less utility as a diagnostic method. An alternative instrumental approach has been purge-and-trap, again followed by GC-MS analysis. A stream of gas (typically helium) is bubbled through the sample to purge out VOCs which are then trapped on an adsorbent bed which is then thermally desorbed for analysis [29]. Unlike SPME this is an exhaustive technique and is quantitative but some preparation of the sample (dilution, addition of reagents) is required. Application has focussed mainly on inflammatory bowel disease [30, 31] and the effects of nutritional supplements [32]. Although not exhaustive, TD-GC-MS is also quantitative but requires little sample preparation while samples can be taken remote from the analytical instrument using simple equipment. Furthermore, in this study it has been used to determine the rate of emission of VOCs from faecal samples rather than to estimate the concentration in the headspace or in the sample matrix itself.

However, all these methods require large, immobile, expensive equipment and have an unacceptably long time-to-result for use as point-of-care diagnostics. Recent studies have begun to address this issue by combining rapid GC separation with a simple sensor for detection [33] or by using a more sophisticated sensor system without a separation step [34]. While successful at identifying CDI, however, these methods are limited by their inability to unequivocally identify individual compounds. A cost-effective means to rapidly quantify specific VOCs in the complex mixture present in faecal headspace would therefore present a significant advance in this field.

Our results must be interpreted with some caution. We were limited to the use of frozen samples, and some loss of VOCs might be expected as a result of the freeze/thaw process. While there is limited evidence that this loss is not great in samples frozen for a few days [10], we have no data on the duration of freezing for the samples that we used. Similarly we have no metadata available on the samples themselves. It is possible that faecal VOC profile will be affected by age, gender and diet while there is evidence of interracial variation (which may of course be related to other factors such as diet and exposure) in sulphides which may or may not extend to the compounds identified in this study [35, 36]. Similarly we were not able to analyse replicate or longitudinal samples so could not assess the extent of inter- and intra- individual variation.

The majority of the VOCs reported here have previously been suggested as potential markers for CDI, but it is notable that some of them were undetectable in a large proportion of our samples. One of them, 5-methyl-2-furancarboxaldehyde, we did not detect at all. Another, 2-(2-Ethoxyethoxy) ethanol, was only found in a handful of samples. This compound is also known as Transcutol and is used for pharmaceutical applications such as topical gels and creams, or as a coating for pills, so is not in any case likely to prove a useful biomarker. Seven other compounds (methyl butanoate, 1H-pyrrole, furan-2-carbaldehyde, 3-methyl butanoic acid, 2-methylbutanoic acid, heptan-4-one and 4-methyl pentanoic acid) were detectable in too few samples to be considered useful. It is not known why these compounds, reported by others, are detected in such a small proportion of our samples. Some compounds were identified in *C*. *difficile* grown in culture and in this case the discrepancy may be the result variation in metabolism of the organism *in vitro* and *in vivo*. With regard to studies utilising stool samples it may relate to different sample treatment or preparation methods.

Of the seven compounds identified as potential biomarkers for CDI, two (3-methylbutanal and dodecane) were suggested by an initial visual inspection of a subset of the data and have not previously been reported so far as we are aware. Emission rate of dodecane was significantly higher in CDI positive samples with and ROC of 0.803. However, dodecane was present in ambient air at higher abundance than in sample headspace so that its use as a practicable marker would be likely to be problematic despite the effectiveness of our equipment at removing it from the sample feed air (see **Table 2**). Since statistical testing on the basis of visual inspection involves something of a circular approach, the validity of 3-methylbutanal as a marker for CDI would require further experimentation.

Propan-1-ol, ethyl propionate and indole were all emitted at significantly higher rates from CDI-positive samples, which accords well with the findings of previous *in vivo* and *in vitro* studies [10, 11, 37]. We have previously reported that these three compounds are also elevated in inflammatory bowel disease, notably in Crohn’s disease [13], and so are possibly reflective of general dysbiosis rather than being specific indicators of CDI. However, these compounds have also emerged as potential CDI biomarkers in studies which have also included IBD patients so their potential utility cannot be dismissed [10, 11].

Hexanoic acid was emitted at a significantly lower rate from CDI-positive samples, nonetheless having a similar ROC to the other potential biomarkers. This contrasts with a study of cultured *C*. *difficile* which concluded that increased production of hexanoic acid, determined by GC, was a potential method for rapid diagnosis [19]. On the other hand our results support the findings of Garner et al. [10] who reported that hexanoic acid was detectable in the headspace of only 9% of stool samples from CDI patients, whereas it was present in >90% of samples from healthy volunteers and patients with *Campylobacter jejeuni* infection and in 61% of patients with ulcerative colitis.

There is anecdotal evidence that stool samples from patients with CDI have a distinctive smell and that experienced nursing staff can distinguish them from non-infected samples. Furthermore, it has been considered that this is due to the presence of 4-methylphenol (*p*-cresol) in samples from patients with CDI. However, the results of formal trials have been inconclusive and it seems likely that nurses in fact use a variety of cues to diagnose potential CDI [38–40]. Animals (for example Cliff the Beagle [41]) with their greater olfactory acuity, have had more success but require housing, feeding and training at significant expense. So far as we are aware this is the first time that increased emission of 4-methylphenol from CDI-positive samples has been demonstrated analytically. 4-methyl phenol is produced by *C*. *difficile* from the catabolism of the aromatic amino acid tyrosine via the 4-hydroxyphenylacetate intermediary [42] and this ability appears to be common to all strains [21]. This pathway may be unique to *C*. *difficile* and is certainly extremely uncommon, with other bacteria metabolising tyrosine to form phenol. The ability to produce 4-methylphenol is probably related to the virulence of *C*. *difficile*, since it continues to grow at concentrations which inhibit other bacteria [43]. There is evidence that the ability to produce and tolerate 4-methyphenol is even more pronounced in hypervirulent strains [44] and it might be possible distinguish these from other strains by their VOC profile [45].

## Conclusions

We investigated whether rapidly-accessible volatile biomarkers of CDI exist in routine stool samples from patients with unexplained diarrhoea. We employed a novel sampling method which allows the rate of release (rather than concentration or abundance) of VOCs to be quantified. This method requires no sample preparation beyond placing it in an appropriate container. A targeted approach was adopted based on published data on the biology and VOC profile of *C*. *difficile* which was successful in identifying seven potential volatile biomarkers for CDI. Of these, 4-methylphenol (*p*-cresol) (AUC ROC curve 0.81) is considered to have considerable promise as it relates closely to the metabolism of *C*. *difficile* and its rate of production may be elevated in hypervirulent strains. Our results show that a volume of faecal headspace gas sampled over a few minutes may contain VOCs indicative of CDI. This approach could be applied to point-of-care diagnosis, though the realisation of a practical method will depend on the development of instrumentation to detect the relevant biomarkers with sufficient rapidity and analytical specificity in the complex mixture of VOCs released by faecal samples.
